# Supplementary material for: Causal interplay between lactose intolerance and gut microbiota: a combined bidirectional Mendelian randomization and in vivo validation study
Source: Front Nutr. 2026 Jun 1;13:1803337. doi: 10.3389/fnut.2026.1803337 (PMC13265576; doi:10.3389/fnut.2026.1803337)
Supplement: Supplementary file 1 [file Data_Sheet_1.zip › supplementary materials/Forward/leave one out cross validation/ebi-a-GCST90027815.finngen_R12_E4_LACTONAS.pdf]

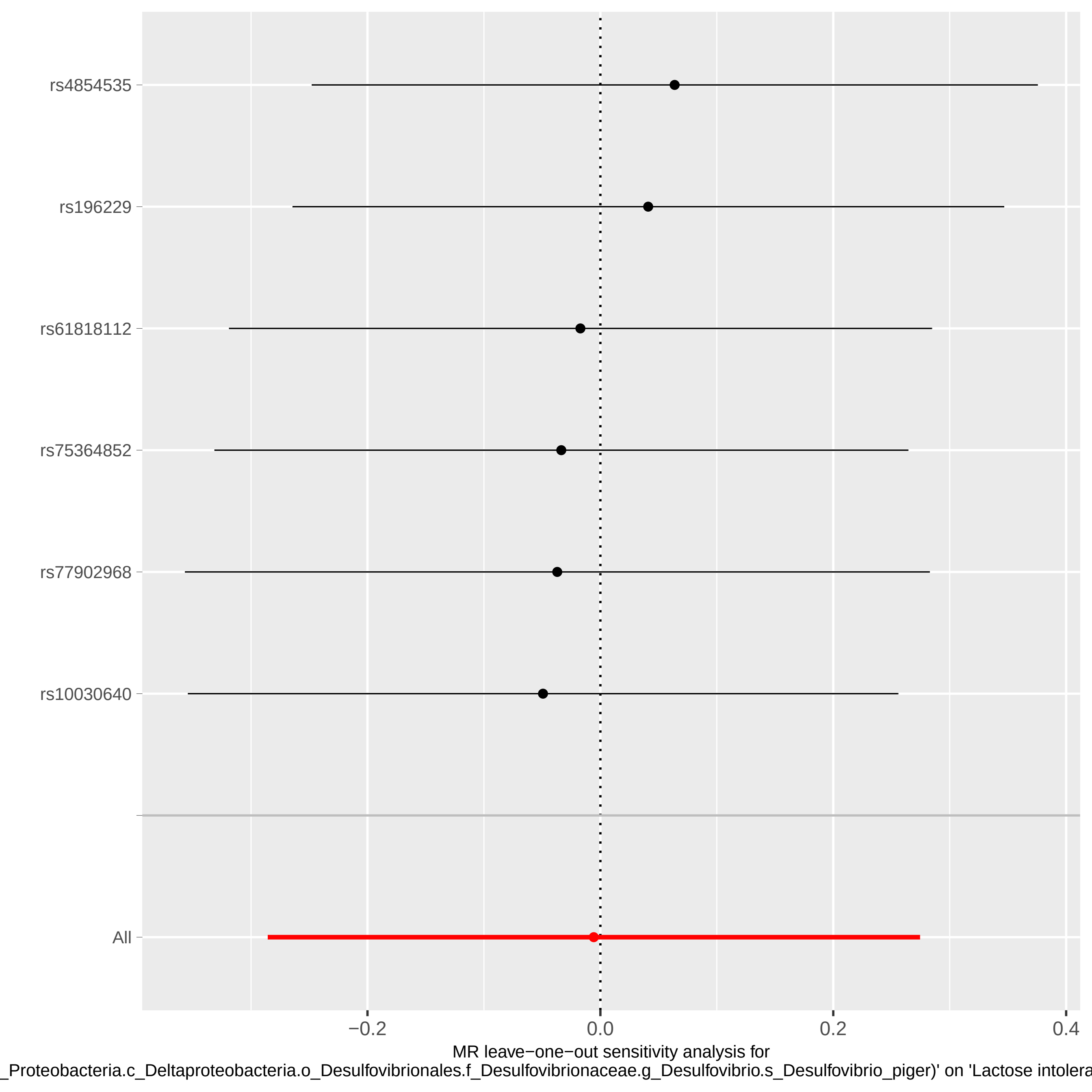

rs4854535
rs196229
rs61818112
rs75364852
rs77902968
rs10030640
All
−0.2
0.0
0.2
0.4
MR leave−one−out sensitivity analysis for
_Proteobacteria.c_Deltaproteobacteria.o_Desulfovibrionales.f_Desulfovibrionaceae.g_Desulfovibrio.s_Desulfovibrio_piger)' on 'Lactose intolera
